# Finnish children’s exposure to paid-for digital food advertising: the pilot study

**DOI:** 10.1017/S1368980026102006

**Published:** 2026-02-16

**Authors:** Peppi Haario, Emma Koivurinta, Päivi Mäki, Maria João Gregório, Kremlin Wickramasinghe, Heli Kuusipalo

**Affiliations:** 1 Department of Public Health, Finnish Institute for Health and Welfare, P.O. Box 30, FI-00271 Helsinki, Finland; 2 Faculty of Nutrition and Food Sciences of the University of Porto, Institute of Public Health, University of Porto, 4050-600 Porto, Portugal; 3 WHO Regional Office for Europe, UN City Marmorvej 51, DK-2100 Copenhagen Ø, Denmark

**Keywords:** Digital food marketing, WHO’s CLICK framework, Marketing for children, Food environment, Paid-for digital food advertising

## Abstract

**Objective:**

To test the ‘Investigate Exposure’ step of the WHO’s CLICK framework and to investigate 12–16-year-old children’s exposure to paid-for digital food advertising in Finland.

**Design:**

The DIGITUTKA pilot study was carried out as part of the EU Joint Action Best-ReMap project. Data on paid-for digital food advertising that children were exposed to via their phones over a two-week period were captured using the RealityMeter application, following the ‘Investigate Exposure’ step of the CLICK framework. Data were collected between April and June 2022 and analysed in Excel, following a protocol outlined by WHO Europe. The WHO Europe Nutrient Profile Model (v1, 2015) was used to determine marketing permission.

**Setting:**

Four schools in Finland

**Participants:**

6th–9th grade students (*n* 34)

**Results:**

Out of the 17 820 captured advertisements, 2316 (13 %) were identified as food or beverage brands and products. The most commonly advertised products were ready-made and convenience foods and composite dishes (16 %, *n* 372) and energy drinks (9 %, *n* 202). The majority of the food and beverage advertisements (*n* 1291, 56 %) were not permitted to be marketed to children, with only one in ten (*n* 222, 9 %) permitted to be marketed to children. A third (35 %) of the food and beverage advertisements could not be identified due to missing information.

**Conclusions:**

Children were exposed to a large number of food and beverage advertisements, most of which were not permitted to be marketed to children. To protect children’s health and prevent obesity, marketing restrictions should be combined with broader changes to the food environment and taxation.

The prevalence of overweight and obesity among children has increased both in Finland^(1,2)^ and globally in recent decades^(3)^. Notably, there has been a significant increase in overweight and obesity among Finnish children over the past three decades^(1,2)^. However, the prevalence of overweight among children aged 2–16 years remained stable between 2020 and 2023^(4)^. Based on the WHO growth reference definitions for children, approximately one in three Finnish girls (28 %) and boys (34 %) aged 12–16 years were living with overweight (including obesity), and 8 % of girls and 14 % of boys were living with obesity, based on measured weight and height^(5)^.

The well-established factors contributing to overweight and obesity in children include a decline in physical activity and a changing food environment that is becoming increasingly obesogenic and health-threatening. Digital food marketing has notably altered the food environment and appears to be one of the main drivers of the rising global obesity epidemic^(6)^. Overweight and obesity affect both the physical and mental health of children, influencing their overall well-being^(7)^. Findings from a systematic review also suggest a weak association between overweight and obesity and lower educational attainment^(8)^. It is a matter of concern that overweight and obesity during childhood are associated with a greater risk of overweight and obesity in adulthood, along with an increased likelihood of developing diseases such as diabetes and CVD at a younger age^(7)^.

The changed global food system produces more affordable and processed food, which is now being marketed more effectively than ever before^(9)^. Children are a significant target group for companies^(10)^. Children encounter food advertising across various channels and formats, predominantly in digital environments, and are exposed daily to digital advertisements for many unhealthy food and beverage products that are high in fat, salt and sugar^(6,11–13)^. Children are a highly vulnerable group, especially prone to the effects of such advertising. It has been observed that adolescents may be even more vulnerable to the marketing of unhealthy food and beverages than younger children^(9)^.

A recent systematic review and meta-analysis^(14)^ highlights that exposure to food marketing significantly influences children’s food consumption, choices, preferences and requests to parents for purchases. Another review published around the same time also found that the digital marketing of unhealthy food and beverages impacts children’s food preferences and intake^(15)^. Food marketing is recognised as negatively influencing children’s eating habits, and exposure to marketing contributes to the development of obesity^(16–20)^. The results indicate that acute exposure to food advertising increases food intake in children, but not in adults^(16)^. A recent study examining the consumption of energy drinks – products that are extensively advertised to children – among Finnish children aged 13 and 15 showed that even infrequent consumption of energy drinks was linked to several negative health indicators^(21)^.

According to a scoping review, food marketing has a similar impact on the consumption of unhealthy foods across both genders^(22)^. Additionally, it has been shown that even adolescents’ critical thinking abilities do not protect them from the effects of marketing^(23)^.

Companies hire celebrities or social media influencers to promote their brands by integrating brand messages into their content^(9)^. Additionally, marketing increasingly involves consumers imitating, sharing or modifying digital advertisements across social platforms^(24)^ – a strategy that has proven highly effective.

Online safety for children is a growing concern in the WHO European Region^(25)^, and restricting food marketing is increasingly recognised as a children’s rights issue^(26)^. This underlines the need to intervene in the obesogenic food environment to prevent the development of obesity and protect children’s well-being. All WHO member states, including Finland, adopted the 2010 resolution to restrict unhealthy food marketing to children^(26)^.

Recent regulations on digital marketing at the EU level, namely the Digital Services Act and the Digital Markets Act, have given significant attention to children. The Digital Services Act and Digital Markets Act form a single set of rules that apply across the EU^(27)^. Their aim is to establish a safer digital environment that protects users’ fundamental rights and ensures a fair competitive landscape for businesses^(27)^. According to the Digital Services Act and Digital Markets Act, targeted advertising must not be directed at children. In addition to the Digital Services Act and Digital Markets Act, Finland is committed to complying with the Revised Audiovisual Media Services Directive (2018/1808/EU)^(28)^. The Audiovisual Media Services Directive creates an EU-level framework to coordinate national legislation across all audiovisual media^(28)^. The directive specifically encourages EU Member States to use co-regulation and self-regulation as tools of intervening in food marketing.

The existing regulations concerning food in Finland include the Food Act (297/2021)^(29)^ and the Consumer Protection Act (No. 38 of 1978)^(30)^. The Consumer Protection Act was the first law to regulate food marketing to children in Finland^(31)^. However, none of the current regulations in Finland prohibit the marketing of unhealthy foods and beverages to children.

A scoping review on food marketing targeted at teenagers found that research on this age group is limited, despite their susceptibility^(32)^. Most studies focus on exposure to television advertisements, while fewer examine food advertising on various digital platforms^(32)^, which are now widely used by teenagers. Additionally, a recent WHO report identified research gaps in food marketing to children, noting that much of the research on its effects has focused on food marketing on television^(33)^.

There is a lack of knowledge regarding the national situation of digital food marketing targeting children. To our knowledge, no previous study in Finland has examined children’s exposure to paid-for digital food advertising by capturing advertisements directly from users’ phones. However, current evidence appears to support the need to reduce and restrict children’s exposure to the digital marketing of unhealthy foods and beverages.

The WHO European Office for the Prevention and Control of Noncommunicable Diseases has developed the CLICK framework to support WHO European Member States in monitoring the digital marketing of unhealthy products targeted at children^(34)^. The CLICK framework provides information on the current situation regarding children’s exposure to digital marketing of unhealthy food and beverage products. The CLICK framework consists of the following five steps: ‘Comprehend the digital system’ (C), ‘Landscape of campaigns’ (L), ‘Investigate exposure’ (I), ‘Capture on-screen’ (C) and ‘Knowledge sharing’ (K). This pilot study explored a tool that implemented the third step of the CLICK monitoring framework, ‘I – Investigate Exposure’. There are various forms of food and beverage marketing in digital media, for example social media marketing, livestreaming, streamers, influencer marketing, influencers and user-generated content^(35)^. Digital marketing is personalised to the user, which makes it important to collect data from children’s devices and to have a method for doing so, as demonstrated in this pilot study. The RealityMeter application used in this pilot study is limited to capturing only paid-for digital advertising from users’ devices.

The aim of this DIGITUTKA pilot study was to collect and analyse data on targeted paid-for digital advertising of unhealthy foods and beverages directed at children aged 12–16 years, as a means of promoting well-being and health. Additionally, the objective was to investigate 12–16-year-old children’s exposure to paid-for digital food advertising in Finland.

## Material and methods

### Participants and sample

The DIGITUTKA pilot study was approved by the Institutional Review Board of The Finnish Institute for Health and Welfare (THL). Research permits for the pilot study were also obtained from the cities of Helsinki and Vantaa, where the children were recruited. For children under the age of fifteen, both the child’s consent and the guardian’s consent were required. A scientific privacy notice – which is a legally required description of the processing of personal data – was provided to the children and guardians participating in the pilot study.

The DIGITUTKA pilot study was carried out as part of the EU Joint Action Best-ReMap project^(31)^ in March and April 2022. The DIGITUTKA used the RealityMeter application, developed by RealityMine, which captures data during smartphone use. There was a contractual relationship between the WHO and RealityMine with RealityMine operating as a subcontractor to the WHO, but there was no contractual link between THL and RealityMine nor between THL and the WHO. There were also ethical considerations that needed to be carefully addressed when installing the RealityMeter application on children’s phones. Therefore, a direct agreement between THL and RealityMine was initiated in July 2021, which required time to prepare and delayed the process. Additionally, THL legal department’s prioritisation of COVID-19-related matters meant that the agreement was not finalised until March 2022.

Children were recruited for the pilot study from four schools in the capital region of Finland – two schools in Helsinki and two in Vantaa. We enrolled 7–26 children per school and received sixty-eight completed consent forms. The final sample comprised thirty-four children, including twenty-two boys and twelve girls. The children were in grades 6–9 and aged between twelve and sixteen years. Among them, 56 % were aged between twelve and fourteen, while 44 % were aged between fifteen and sixteen.

Data collection for the final sample (*n* 34) was carried out over a period of 1–15 d in April and June 2022 using the RealityMeter application. Since the RealityMeter application was only compatible with Android phones, we were able to recruit only children who had Android phones.

### Data collection

The principal of each participating school received an individual pilot study information sheet. Parents were informed about the research through a video distributed via the Wilma message service, which is the web interface for the educational institution’s administration system. A short informational video was also shown to all children in the classroom prior to recruitment. Additionally, project researchers distributed information sheets, consent forms, and the pilot study background questionnaire to the classes. Both children and parents received their own information sheets and consent forms. We received sixty-eight completed consent forms, but only half of them (50 %) successfully used the RealityMeter application. Some of the children had problems downloading the application, and in some cases, the activation code did not work. Children participating in the pilot study (*n* 34) had the option to complete the background questionnaire together with their parents. For children under fifteen years of age, parental consent was required in addition to the child’s consent. The consent and background information forms were collected by the teacher, and the completed forms were retrieved from the schools. The consent and background questionnaires included information such as the subject’s name, gender, age, telephone number, email address, school name, school class, guardian’s name and contact details. After the study, the children received a cinema ticket as a thank-you gift.

## Methods

The method used in the DIGITUTKA pilot study was based on the CLICK framework for mapping digital food marketing targeted at children.

The CLICK framework is a five-step process designed to collect data on children’s exposure to marketing^(34)^. The third step of CLICK framework is ‘Investigate exposure’, which was used in this pilot study to map exposure to paid-for digital advertising experienced by 12–16-year-old children. This was done using an installed smartphone app that, with consent, monitors and aggregates data on children’s interactions with advertisements on certain websites and social media platforms^(34)^.

The RealityMeter application, developed by RealityMine, captures data during smartphone use. Instructions on how to install and download the RealityMeter application were provided to the children at school as well as to their parents. Only children with Android phones (such as Samsung, OnePlus, Huawei, Honor, Nokia, Xiaomi or Motorola) were able to participate in the pilot study. Therefore, children with iPhones could not participate because the RealityMeter application would have been more difficult to set up on iPhones and the advertisements could only be captured on YouTube. In this DIGITUTKA pilot study, advertisements were captured from Facebook, Twitter, Snapchat, TikTok, YouTube and Instagram.

The RealityMeter application provided the client key, panellist ID, media IDs for each advertisement (a code that identifies the exact advertisement captured by the device), provider name, location, advertisement title, social media platform where the advertisement was seen, a scan of the social media platform’s contents, surreptitious advertisement in videos, advertisements shown before, between or after videos, banner advertisements when pausing videos or during other activities, the duration of advertisement video, time spent viewing each advertisement (the time spent on the URL-address in seconds), the number of advertisements clicked by the child and the type of advertisement (whether it was paid – funded by advertisers – or user-generated – shared content).

Food and beverage advertisements were classified using the first WHO Europe Nutrient Profile Model (NPM, v1 2015), developed by the WHO Regional Office for Europe to restrict and monitor the marketing of foods and beverages to children^(36)^. The WHO Europe NPM is a tool for categorising unhealthy food and beverage products that are high in fat (total and saturated), salt and sugar (total and added) and as well as those containing non-sugar sweeteners. First, all the advertisements captured by the RealityMeter were categorised as either food and beverage advertisements or other advertisements. All the food and beverage products were classified into the following categories^(36)^: (1) chocolate and sugar confectionery, energy bars, and sweet toppings and desserts, (2) cakes and sweet biscuits (cakes (and) sweet biscuits and pastries; other sweet bakery wares; and dry-mixes for making such), (3) savoury snacks, (4) beverages (subcategories: juices, milk drinks, plant-based milks, energy drinks, soft drinks, bottled waters and other drinks), (5) edible ices, (6) breakfast cereals, 7) yoghurt, sour milk, cream and other similar foods, (8) cheese, (9) ready-made and convenience foods and composite dishes, (10) butter, other fats and oils, (11) bread, bread products and crisp breads, (12) fresh or dried pasta, rice and grains, 13) fresh and frozen meat, poultry, game, fish and similar, (14) processed meat, poultry, game, fish and similar, (15) fresh and frozen fruit, vegetables and legumes, (16) processed fruit and vegetables, (17) plant-based foods/ meat analogues and (18) sauces, dips and dressings.

Nutritional information, such as total fat (g/100 g), saturated fat (g/100 g), total sugars (g/100 g), added sugars (g/100 g), non-sugar sweeteners (g/100 g), salt (g/100 g) and energy (kcal/100 g), was collected from grocery stores’ websites for all food and beverage products. The WHO Europe NPM includes category-specific nutritional thresholds for each food and beverage product, which were provided for all categories in the model (except fruit and vegetables). All food and beverage products were defined as either permitted or not permitted to be marketed to children based on their nutritional thresholds using the WHO Europe NPM.

### Statistical analysis

Statistical analyses were conducted using Excel following a protocol outlined by WHO Europe. The total number of advertisements was tabulated. Food and beverage advertisements captured from different platforms were described using frequencies. Food and beverage advertisements are defined as either permitted or not permitted to be marketed to children based on the nutritional information of the products, according to the WHO Europe NPM. Additionally, all food or beverage brands appearing in the advertisements were identified.

## Results

In total, 17 820 advertisements were captured on the smartphones of the thirty-four participating children during the two-week study period. Of these 17 820 advertisements, 2316 (13 %) were identified as food or beverage brands and products.

**Figure 1.**
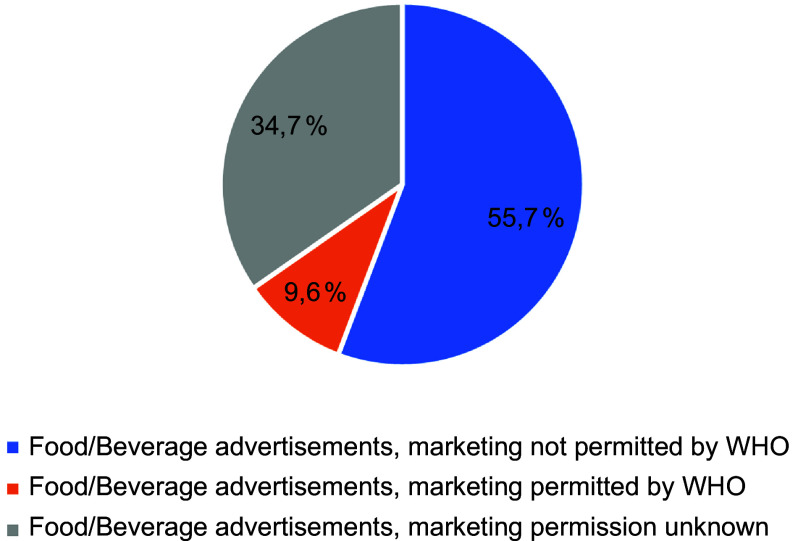
The proportion of food and beverage advertisements (*n* 2316) permitted or not permitted to be marketed to children according to WHO Europe Nutrient Profile Model, NPM (the DIGITUTKA pilot study, children aged 12–16 years).

**Figure 2.**
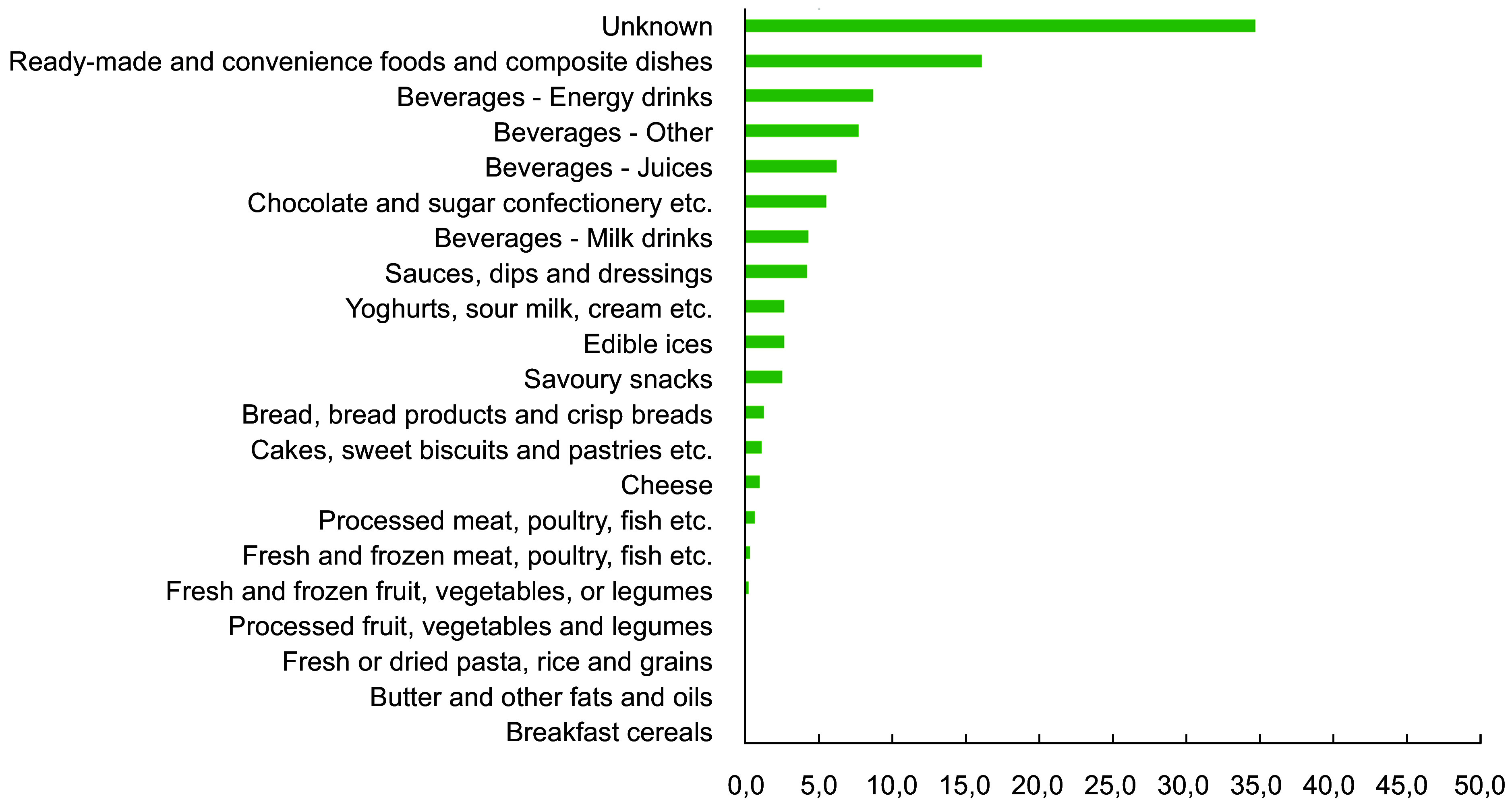
The proportion (%) of captured food and beverage advertisements (*n* 2316) according to the WHO Europe Nutrient Profile Model, NPM (the DIGITUTKA pilot study, children aged 12–16 years).

All food and beverage product advertisements (*n* 2316) were categorised according to the WHO Europe NPM food categories. Figure 1 shows the proportion of captured food and beverage advertisements according to WHO Europe NPM food categories. Of the 2316 food and beverage advertisements, the most frequently advertised products were ready-made and convenience foods and composite dishes (16 %, *n* 372), energy drinks (9 %, *n* 202), other beverages (8 %, *n* 179), juices (6 %, *n* 144) and chocolate and sugar confectionery, energy bars, sweet toppings and desserts (6 %, *n* 128) (Figure 2). The exact content of 35 % of food and beverage product advertisements remained unknown. The WHO Europe NPM category ‘beverages’ was divided into four groups – energy drinks, juices, milk drinks and other drinks like sodas – to represent different types of beverages. Only a few advertisements were captured from the categories of ‘processed fruit, vegetables and legumes’ (*n* 1), ‘fresh and frozen fruit, vegetables, or legumes, etc.’ (*n* 6) and ‘fresh and frozen meat, poultry, fish, etc.’ (*n* 8). No advertisements were captured from the categories of ‘breakfast cereals’, ‘butter and other fats and oils’, and ‘fresh or dried pasta, rice and grains’.

**Figure 3.**
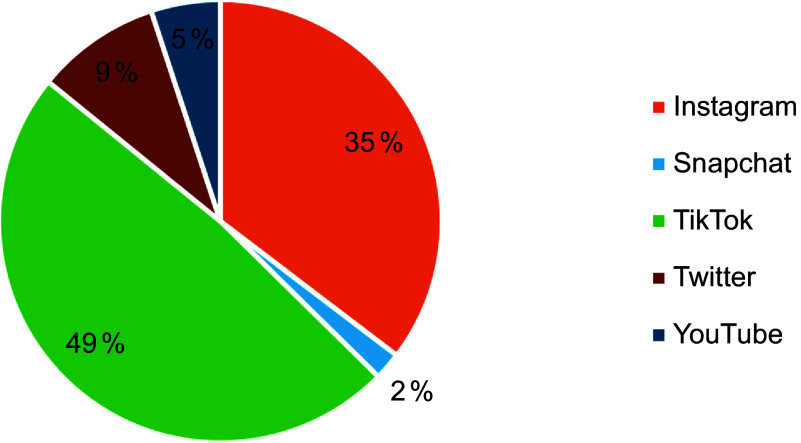
The distribution of food and beverage advertisements on different social media platforms (the DIGITUTKA pilot study, children aged 12–16 years).

The RealityMeter captured advertisements from six different platforms: Facebook, Twitter, Snapchat, TikTok, YouTube and Instagram. Most of the food and beverage advertisements were captured from TikTok (49 %) and Instagram (35 %) which can be explained by their popularity among children. Every tenth (9 %) food and beverage advertisement was captured from Twitter (Figure 3). The most frequently appearing food or beverage advertisements were produced by Lidl (a discount supermarket chain), Wolt (a delivery service), Valio and Fazer (food industry companies) and McDonald’s.

According to the WHO Europe NPM, food and beverage advertisements are classified as either permitted or not permitted to be marketed to children based on the nutritional information of the products. In this study, more than half of the food and beverage advertisements (*n* 1291, 56 %) were not permitted to be marketed to children. In contrast, only one in ten (*n* 222, 9 %) food and beverage advertisements were permitted to be marketed to children, according to the WHO Europe NPM (Figure 1). A third of food and beverage product advertisements had unknown marketing permission due to missing MediaIDs in the data (Figure 1). Without the MediaID, it was not possible to verify the exact content of the advertised food or beverages products.

## Discussion

This pilot is one of the very few studies in the world conducted in collaboration with national institutions, providing valuable insights for other countries. The overall aim of the pilot was to test the feasibility of using a metadata collection tool, the RealityMeter, to implement the ‘Investigate Exposure’ step of the WHO’s CLICK framework and to demonstrate the importance of monitoring of paid-for digital advertising targeted at children. The aim was also to investigate 12–16-year-old children’s exposure to one form of digital food marketing in Finland: paid-for digital advertising.

The main findings, categorised by topic, were as follows.

### Technical feasibility

We encountered technical problems with downloading the RealityMeter and missing MediaIDs in the data. Due to a large number of missing MediaIDs (35 %), the RealityMeter could not provide accurate information on children’s exposure to paid-for digital advertising of unhealthy food and beverage products.

### Accuracy and comprehensiveness of data generated by the RealityMeter application

It is notable that the RealityMeter application is limited to paid-for digital advertising^(34)^ and is not able to capture, for example, influencer marketing or brand-owned social media content. An Australian study, in which children recorded their mobile device screens for 2 weekdays and 1 weekend day, showed that 24 % of the viewed advertisements were paid-for advertisements^(37)^. Assuming that the pattern in Finland is quite similar, it means that the RealityMeter could have detected approximately one quarter of all viewed advertisements. Especially considering that 35 % of the viewed food and beverage advertisements could not be identified, it is not feasible to estimate the actual number of food and beverage advertisements seen. Nevertheless, it is clear that Finnish children aged 12–16 years are consistently exposed to a large amount of digital food marketing in various forms. Furthermore, majority of food and beverage advertisements seen by children were products high in fat, salt and sugar, which are not permitted to be marketed to children according to the WHO Europe NPM.

### Exposure to paid-for food marketing

In this pilot study, majority of food and beverage advertisements (56 %) were not permitted to be marketed to children. Only 9 % of the food and beverage advertisements that could be identified were permitted to be marketed to children, according to the WHO Europe NPM. However, it is important to note that 35 % of food and beverage advertisements could not be identified due to missing MediaIDs in the data.

In similar studies, majority of advertisements seen by children were not permitted to be marketed to them^(37–40)^. The ‘Investigate Exposure’ step of the WHO’s CLICK framework used in this pilot study was also implemented in the Norwegian and Portuguese pilot studies. A Norwegian pilot study found that eight out of ten food and beverage advertisements were not permitted to be marketed to children, according to the WHO Europe NPM^(38)^. The Portuguese pilot study found that 61 % of the food products featured in the posts were identified as food and/or beverage brands and products, and almost 86 % of them were not permitted according to the Portuguese NPM, which is considered in the Portuguese law that restricts food marketing to children^(41)^.

A pilot study conducted in the USA and Canada^(39)^ found that most or almost all (97 %–100 %) of food advertisements identified in movie theatres were considered not permitted to be marketed to children according to the WHO Europe NPM. These findings were in line with the results of a Mexican study, which used the ‘Comprehend the digital system’ step of WHO’s CLICK framework. The study showed that 93 % of the food promoted in posts appealing to children or adolescents was classified as unhealthy food according to the Mexican warning labels nutrient profile^(40)^. It should be noted that the data from the Mexican study were not collected from children’s own devices. An Australian study, in which children were asked to give consent to video record their mobile device screens for 2 weekdays and 1 weekend day, also showed that most of the food advertisements seen by children on the Internet via mobile devices were not permitted to be marketed to children, according to WHO Europe NPM^(37)^.

Our findings suggest that energy drinks were among the most frequently advertised food and beverage products to children in Finland. A recent study examined the consumption of energy drinks among Finnish children and highlighted the importance of a rigorous evaluation of energy drink marketing. The results of that study showed that even infrequent consumption of energy drinks was linked to several negative health indicators among children^(21)^. The Organisation for Economic Co-operation and Development has estimated that restricting the advertising of unhealthy foods and beverages, as part of effective policy strategies, helps prevent obesity in children^(42)^. A similar conclusion was reached in a report on the food environment of Finnish children, which found that restricting marketing is cost-effective, especially when targeted at children over 12 years old in Finland^(43)^. Also, the findings from marketing of unhealthy foods to children (EPELI) project clearly support stricter and more restrictive regulations on marketing, particularly to protect 13–17-year-old youths^(44)^.

### Exposure to paid-for unhealthy food marketing on different platforms

In this pilot study, most of paid-for food and beverage advertisements were captured from TikTok and YouTube. Almost half of paid-for food and beverage advertisements were captured from TikTok (49 %). In contrast, a study examining the power and platforms of food marketing,^(45)^ using a mobile app called GrabFM! (Grab Food Marketing!), found that Instagram was a top source of food marketing content among Canadian teenagers. In another Canadian study utilising the same data and the GrabFM! app, majority of all advertisements (72 %) seen by teenagers were for branded beverages, fast food, and candy/chocolate^(46)^, which is broadly similar to the findings of this pilot study. YouTube, TikTok, and Instagram are popular applications among children, which may explain why most food and beverage advertisements are seen through these applications. It is also possible that food and beverage advertisers choose to promote more of their products on these platforms than on others, due to their popularity among children.

A Canadian study found that use of different social media platform did not vary by gender. Instagram emerged as the most popular platform for viewing advertisements among both genders. However, TikTok and Snapchat were also notable popular, particularly among girls^(47)^. Interestingly, another Canadian study found that food and beverage companies advertise different products to girls and boys on social media^(48)^, indicating that advertising targeting is not only directed at children in general but also differentiated by gender. Due to the small number of participants, this pilot study was not able to examine gender differences.

### The current state of food marketing to children in Finland

Our food environment has changed significantly due to digital food marketing, which appears to be one of the major drivers of the global obesity epidemic^(6)^. Exposure to food marketing has a significant impact on children’s food consumption, choices, preferences and purchase requests directed at parents^(14)^. A new policy document published by the Food Research Collaboration on food marketing in the UK underlines that the substantial evidence supports the need to shift towards healthier and more sustainable diets to protect both human and planetary health. However, the current food environment does not support this transition^(49)^. Restricting food marketing is one way to prevent obesity among children, alongside excise taxes and clear food labelling. Across Europe, the priority should be to invest in changing the food environment.

The marketing of unhealthy foods and beverages to children is not prohibited by law in Finland. A new guideline on the marketing of unhealthy foods targeted at children was published at the end of 2024^(50)^, after this pilot study. To support-decision-making, we need to monitor Finnish children’s nutrition and their exposure to digital marketing – beyond paid-for marketing – through long-term research. The Board of Trading Practices in the Food Supply Chain was established in connection with the Finland Chamber of Commerce in 2023. The task of that Board is to evaluate food marketing and support companies in developing and improving their self-regulation and nutritionally responsible marketing. Mandatory regulation, such as health-related taxes, should be considered to tackle obesity. In the future, it is crucial to consider children’s opinions and involve them in the decision-making process.

### Methodological considerations

A major strength of the pilot study lies in its ability to provide more accurate data by capturing advertisements directly from users’ phones.

Some limitations need to be acknowledged. The main limitation was that the RealityMeter is only able to capture paid-for digital advertising. Additionally, the RealityMeter application was only available for Android phones. Several participants were hesitant to admit during the recruitment process that they did not own an iPhone. In addition, some of the children experienced difficulties downloading the application, and in some cases, the activation code did not work. We also faced challenges in fetching data. These limitations may have led to a bias, especially considering that iPhone users were unable to participate in the pilot study at all. While the RealityMeter application is suitable for research, it is quite challenging to use in studies involving a large number of children. Also, the legal processes required for deploying the RealityMeter application were time-consuming, as was obtaining consent from both children and their parents.

Additionally, 35 % of food and beverage product advertisements had an unknown marketing permission status due to missing MediaIDs in the data. However, we observed that some of the advertisements with missing MediaIDs were from brands that exclusively offer products not permitted to be marketed to children, such as Coca-Cola Company. We do not know whether MediaIDs were available only for a certain period or if they were missing from the beginning. Also, the Norwegian pilot study encountered similar problems with the RealityMeter application^(38)^. More challenging were brands that offer both healthy and unhealthy food and beverage products, such as supermarkets or bakeries. This may have led to an under-representation of healthy food and beverage advertisements in the results. We were also unable to obtain the variable indicating the duration of time spent watching advertisements. In general, the high non-response rate and the challenges related to fetching and analysing data are potential sources of bias.

### Conclusions

These results indicate that policy-level actions are needed, as current global measures to prevent obesity are insufficient. The pilot study highlighted the need to monitor digital food marketing targeted at children. Due to the limitations of the RealityMeter application, future monitoring of digital food marketing to children should be complemented with additional methods to achieve a more comprehensive overview. Monitoring should be implemented in a comparable manner, and further methodological development is needed. In the future, it will be necessary to implement effective measures, such as legislation or regulation, to restrict the marketing of unhealthy foods and beverages to children and thereby reduce their exposure to such marketing, ensuring that children’s rights are respected. Policymakers should ensure that regulations restricting the marketing of unhealthy foods and beverages protect children’s health and help prevent obesity. To achieve the greatest impact on children’s food and beverage consumption, changes to the entire food environment are needed, along with the implementation of taxation on unhealthy foods and sugary beverages – while also taking into account the pricing of healthy foods. Mandatory front-of-package nutrition labelling, indicating the nutritional quality of foods and beverages, should be introduced concurrently with marketing restrictions in Finland.

The authors alone are responsible for the views expressed in this publication and such views do not necessarily represent the views, decisions or policies of the institutions with which they are affiliated.

## References

[1] Finnish Institute for Health and Welfare (THL) (2023) Prevalence of overweight and obesity of children and adolescents 2022. https://sampo.thl.fi/pivot/prod/en/finlapset/growth/fact_growth (accessed March 2024).

[2] Vuorela N , Saha MT & Salo MK (2011) Change in prevalence of overweight and obesity in Finnish children – comparison between 1974 and 2001. Acta Paediatr 100, 109–115. 20712840 10.1111/j.1651-2227.2010.01980.x

[3] NCD Risk Factor Collaboration (NCD-RisC) (2017) Worldwide trends in body-mass index, underweight, overweight, and obesity from 1975 to 2016: a pooled analysis of 2416 population-based measurement studies in 128·9 million children, adolescents, and adults. Lancet 16, 2627–2642. 10.1016/S0140-6736(17)32129-3 PMC5735219 29029897

[4] Finnish Institute for Health and Welfare (THL) (2023) Prevalence of Overweight and Obesity of Children and Adolescents 2023. FinChildren Register Monitoring. https://www.thl.fi/finlapset_ylipainoraportti/en/index.html (accessed December 2023).

[5] Finnish Institute for health and welfare (THL) (2023) Register of Primary Health Care visits – THL. https://thl.fi/en/statistics-and-data/data-and-services/register-descriptions/register-of-primary-health-care-visits (accessed December 2023).

[6] Boyland E (2025) Would reducing children’s exposure to food advertising prevent unhealthy weight gain? Curr Obes Rep 14, 55. 40542916 10.1007/s13679-025-00648-6 PMC12182458

[7] Sahoo K , Sahoo B & Choudhury AK et al. (2015) Childhood obesity: causes and consequences. J Family Med Prim Care 4, 187–192. 25949965 10.4103/2249-4863.154628 PMC4408699

[8] Caird J , Kavanagh J , O’Mara-Eves A et al. (2014) Does being overweight impede academic attainment? A systematic review. Health Educ J 73, 497–521.

[9] Harris JL , Yokum S & Fleming-Milici F (2021) Hooked on junk: emerging evidence on how food marketing affects adolescents’ diets and long-term health. Curr Addict Rep 8, 19–27.

[10] Wided B (2021) Youth Marketing to Digital Natives, pp. 1–288. Cheltenham: Edward Elgar Publishing.

[11] Norman J , Kelly B , Boyland E et al. (2016) The impact of marketing and advertising on food behaviours: evaluating the evidence for a causal relationship. Curr Nutr Rep 5, 139–149.

[12] Potvin Kent M , Pauzé E , Roy EA et al. (2019) Children and adolescents’ exposure to food and beverage marketing in social media apps. Pediatr Obes 14, e12508. 30690924 10.1111/ijpo.12508 PMC6590224

[13] Gascoyne C , Scully M , Wakefield M et al. (2021) Food and drink marketing on social media and dietary intake in Australian adolescents: findings from a cross-sectional survey. Appetite 166, 105431. 34062174 10.1016/j.appet.2021.105431

[14] Boyland E , McGale L , Maden M et al. (2022) Association of food and nonalcoholic beverage marketing with children and adolescents’ eating behaviors and health: a systematic review and meta-analysis. JAMA Pediatr 176, e221037. 35499839 10.1001/jamapediatrics.2022.1037 PMC9062773

[15] Mc Carthy CM , de Vries R & Mackenbach JD (2022) The influence of unhealthy food and beverage marketing through social media and advergaming on diet-related outcomes in children-a systematic review. Obes Rev 23, e13441. 35301815 10.1111/obr.13441 PMC9286387

[16] Boyland EJ , Nolan S , Kelly B et al. (2016) Advertising as a cue to consume: a systematic review and meta-analysis of the effects of acute exposure to unhealthy food and nonalcoholic beverage advertising on intake in children and adults. Am J Clin Nutr 103, 519–533. 26791177 10.3945/ajcn.115.120022

[17] Coates AE , Hardman CA , Halford JCG et al. (2019) Social media influencer marketing and children’s food intake: a randomized trial. Pediatrics 143, e20182554. 30833297 10.1542/peds.2018-2554

[18] Halford JC , Boyland EJ , Hughes GM et al. (2008) Beyond-brand effect of television food advertisements on food choice in children: the effects of weight status. Public Health Nutr 11, 897–904. 18005487 10.1017/S1368980007001231

[19] Lobstein T & Dibb S (2005) Evidence of a possible link between obesogenic food advertising and child overweight. Obes Rev 6, 203–208. 16045635 10.1111/j.1467-789X.2005.00191.x

[20] Sadeghirad B , Duhaney T , Motaghipisheh S et al. (2016) Influence of unhealthy food and beverage marketing on children’s dietary intake and preference: a systematic review and meta-analysis of randomized trials. Obes Rev 17, 945–959. 27427474 10.1111/obr.12445

[21] Puupponen M , Tynjälä J , Välimaa R et al. (2023) Associations between adolescents’ energy drink consumption frequency and several negative health indicators. BMC Public Health 6, 258. 10.1186/s12889-023-15055-6 PMC9903583 36747163

[22] Castronuovo L , Guarnieri L , Tiscornia MV et al. (2021) Food marketing and gender among children and adolescents: a scoping review. Nutr J 20, 52. 34099000 10.1186/s12937-021-00706-4 PMC8186097

[23] Packer J , Croker H , Goddings AL et al. (2022) Advertising and young people’s critical reasoning abilities: systematic review and meta-analysis. Pediatrics 50, e2022057780. 10.1542/peds.2022-057780 PMC9724173 36377381

[24] Kabir H , Sultana S , Hossain M et al. (2025) The impact of digital marketing strategies on consumer behavior: a comprehensive review. Bus Soc Sci 3, 1–8.

[25] World Health Organization (2024) Making the WHO European Region the Healthiest Online Environment for Children: Position Statement. https://www.who.int/andorra/publications/m/item/making-the-who-european-region-the-healthiest-online-environment-for-children-position-statement (accessed March 2024).

[26] World Health Organization (2010) Set of Recommendations on the Marketing of Foods and Non-Alcoholic Beverages to Children. https://iris.who.int/server/api/core/bitstreams/6ad6b30a-57e0-4193-8515-6e8d9f9545e1/content (accessed March 2024).

[27] European Commission (2022) The Digital Services Act Package | Shaping Europe’s Digital Future. https://digital-strategy.ec.europa.eu/en/policies/digital-services-act-package (accessed December 2023).

[28] European Commission (2018) Revision of the Audiovisual Media Services Directive (AVMSD) | Shaping Europe’s Digital Future. https://digital-strategy.ec.europa.eu/en/policies/revision-avmsd (accessed December 2023).

[29] Oy EP & FINLEX® (2022) Food Act 297/2021. https://www.finlex.fi/en/legislation/translations/2021/eng/297 (accessed December 2023).

[30] Oy EP & FINLEX® (2024) Consumer Protection Act 38/1978. https://www.finlex.fi/fi/lainsaadanto/saadoskaannokset/1978/eng/38 (accessed December 2023).

[31] Best-ReMaP (2023) A Europe-Wide Joint Action, Healthy Food for a Healthy Future (2020–2023) https://bestremap.eu/results/ (accessed December 2023).

[32] Truman E & Elliott C (2019) Identifying food marketing to teenagers: a scoping review. Int J Behav Nutr Phys Act 16, 67. 31426809 10.1186/s12966-019-0833-2 PMC6700978

[33] World Health Organization (2023) Policies to Protect Children from the Harmful Impact of Food Marketing: WHO Guideline. https://www.who.int/publications-detail-redirect/9789240075412 (accessed March 2024). 37440677

[34] World Health Organization (2021) Monitoring and Restricting Digital Marketing of Unhealthy Products to Children and Adolescents: Report Based on the Expert Meeting on Monitoring of Digital Marketing of Unhealthy Products to Children and Adolescents: Moscow, Russian Federation, June 2018. https://www.who.int/europe/publications/i/item/WHO-EURO-2019-3592-43351-60815 (accessed December 2023).

[35] Maksi SJ , Keller KL , Dardis F et al. (2023) The food and beverage cues in digital marketing model: special considerations of social media, gaming, and livestreaming environments for food marketing and eating behavior research. Front Nutr 10, 1325265. 38384857 10.3389/fnut.2023.1325265 PMC10880034

[36] World Health Organization (2015) WHO Regional Office for Europe Nutrient Profile Model. https://www.who.int/publications-detail-redirect/WHO-EURO-2015-6894-46660-67850 (accessed December 2023).

[37] Kelly B , Bosward R & Freeman B (2021) Australian children’s exposure to, and engagement with, web-based marketing of food and drink brands: cross-sectional observational study. J Med Internet Res 23, e28144. 34255675 10.2196/28144 PMC8314155

[38] Consumption Research Norway (SIFO) & OsloMet (2020) Mapping the Landscape of Digital Food Marketing: Investigating Exposure of Digital Food and Drink Advertisements to Norwegian Children and Adolescents. https://painelobesidade.com.br/biblioteca/mapping-the-landscape-of-digital-food-marketing-investigating-expusure-of-digital-food-and-drink-advertisements-to-norwegian-children/ (accessed March 2024).

[39] Wong S , Pauzé E , Hatoum F et al. (2020) The frequency and healthfulness of food and beverage advertising in movie theatres: a pilot study conducted in the United States and Canada. Nutrients 12, 1253. 32354061 10.3390/nu12051253 PMC7282003

[40] Valero-Morales I , Nieto C , García A et al. (2023) The nature and extent of food marketing on Facebook, Instagram, and YouTube posts in Mexico. Pediatr Obes 18, e13016. 36867060 10.1111/ijpo.13016

[41] Gaspar MAB (2022) Assessment of Social Media Content of the Most Marketed Food and Beverages Brands: WHO CLICK Portuguese Pilot Study. https://repositorio-aberto.up.pt/bitstream/10216/144110/2/582325.pdf (accessed November 2023).

[42] Organisation for Economic Co-operation and Development (2019) The Heavy Burden of Obesity: The Economics of Prevention. https://www.oecd.org/els/the-heavy-burden-of-obesity-67450d67-en.htm (accessed December 2023).

[43] Rantala E , Martikainen J , Lakka T et al. (2020) Healthy food environment for Finnish children and adolescents: the current state and policy recommendations for improving it. Publications of the Government‘s analysis, assessment and research activities 2020:19. Helsinki, Finland: The Prime Minister’s Office. https://urn.fi/URN:ISBN:978-952-287-929-5 (accessed December 2023).

[44] Fogelholm M , Närvänen E , Erkkola M et al. (2021) Marketing of unhealthy foods to children and youth: Situation in Finland and rules for regulation. Publications of the Government’s analysis, assessment and research activities 2021:57. Helsinki, Finland: The Prime Minister’s Office. http://urn.fi/URN:ISBN:978-952-383-170-4 (accessed March 2024).

[45] Elliott C , Truman E & Aponte-Hao S (2022) Food marketing to teenagers: examining the power and platforms of food and beverage marketing in Canada. Appetite 173, 105999. 35292304 10.1016/j.appet.2022.105999

[46] Elliott C , Truman E & Black JE (2023) Tracking teen food marketing: participatory research to examine persuasive power and platforms of exposure. Appetite 186, 106550. 37019155 10.1016/j.appet.2023.106550

[47] Elliott CD & Truman E (2024) Food marketing on digital platforms: what do teens see? Public Health Nutr 27, e48. 38269541 10.1017/S1368980024000235 PMC10882529

[48] Amson A , Pauzé E , Remedios L et al. (2023) Adolescent exposure to food and beverage marketing on social media by gender: a pilot study. Public Health Nutr 26, 33–45. 36321517 10.1017/S1368980022002312 PMC11077454

[49] Haffner T & Culliford A (2023) The Food Marketing Environment: A Force for or Against Human and Planetary Health? Food Research Collaboration Policy Insight. ISBN: 978-1-903957-80-6. https://foodresearch.org.uk/publications/the-food-marketing-environment-a-force-for-or-against-human-and-planetary-health/ (accessed December 2023).

[50] Ministry of Social Affairs and Health (2024) Marketing of Food to Children – Guidelines for Protecting Children from the Harmful Effects of Marketing. https://lapsistrategia.fi/wp-content/uploads/2024/12/Ohjeistus_Elintarvikkeiden-markkinointi-lapsille_16-12-2024-4.pdf (accessed February 2025).

